# Effect of lipopolysaccharide on the characteristics of endothelial progenitor cells from bone marrow in mice

**DOI:** 10.3892/mmr.2013.1833

**Published:** 2013-11-28

**Authors:** HAO LI, YONG QIANG, LIAN WANG, CANHUI LIU, NAN YANG, LEI XIONG, JUN YI, HUA JING, HAIWEI WU

**Affiliations:** Department of Cardiothoracic Surgery, Jinling Hospital, Clinical Medicine School of Nanjing University, Nanjing, Jiangsu 210002, P.R. China

**Keywords:** lipopolysaccharide, acute lung injury, endothelial progenitor cells

## Abstract

Previous studies have shown that lipopolysaccharide (LPS) induces acute lung injury (ALI), and that endothelial progenitor cells (EPCs) participate in tissue repair. Therefore, in this study it was hypothesized that LPS influences the number and function of EPCs directly. In order to investigate this, an *in vitro* study was performed using EPCs. EPCs were cultured for seven days (early EPCs), and then treated with increasing concentrations of LPS (10 pg/ml, 100 pg/ml, 1 ng/ml, 10 ng/ml and 100 ng/ml) for 4, 8, 12, and 24 h. The proliferation, senescence and adhesion of EPCs was then assessed. Alongside this an *in vivo* study was also performed. Mice were administered LPS (2.5 mg/kg) via the trachea. After 4, 8, 12, and 24 h, EPCs were harvested and cultured for seven days, and the proliferation, senescence and adhesion of the EPCs were examined. The results showed that the rate of adhesion and senescence of EPCs decreased *in vitro* when treated with 10 and 100 ng/ml LPS. The adhesion and senescence rate also decreased after 12 and 24 h *in vivo*. Proliferation, however, was increased *in vitro* following treatment with 10 and 100 ng/ml LPS, but proliferation *in vivo* decreased after 8 and 12 h. The effects of LPS on EPCs were distinct *in vivo* and *in vitro. In vitro,* cells were sensitive to 100 ng/ml LPS. In the course of ALI induced by LPS, the proliferation and adhesion activity of the EPCs was activated in 8 h and then gradually decreased with time.

## Introduction

Acute lung injury (ALI) and the more severe form, acute respiratory distress syndrome (ARDS), are life-threatening respiratory failures that are caused by numerous factors (1). Despite the advances in treatment, ALI/ARDS remains a serious problem due to its high mortality (<40%) and morbidity rates (2). ARDS is characterized pathologically by diffuse alveolar damage, alveolar capillary leakage and protein-rich pulmonary edema, leading to the clinical manifestations of poor lung compliance, severe hypoxemia and bilateral infiltrates being observed on chest radiographs (1). A study of critical care units in the USA in 2005 estimated the incidence of ARDS to be 58/100,000 individuals with 141,500 novel cases and an annual death rate of 59,000 per year (3). Several drugs have been investigated in the treatment of ALI/ARDS; however, to date, none of these have been successful (4).

Circulating endothelial progenitor cells (EPCs) were first discovered by Asahara *et al* in 1997 (5), and they have since been found to have a role in neovascularization and vasculogenesis (6). In ALI, an increase in the number of circulating EPCs is associated with an improved survival rate (7). Lam *et al* (8) demonstrated that autologous transplantation of EPCs preserves pulmonary endothelial function and maintains the integrity of the pulmonary alveolar-capillary barrier. Therefore, transplantation of EPCs may be a novel, cell-based, endothelium-targeted therapeutic strategy for the treatment and prevention of ALI/ARDS (8). Studies in rats have found that administering EPCs results in an increase in the number of EPCs targeted to injured lung tissue (9–11), which significantly mitigates lung injury and improves survival of ALI rats (10,11).

Lipopolysaccharide (LPS), a major component of the cell membrane of Gram-negative bacteria, has a critical role in ALI and ARDS. LPS induces ALI and this has been widely used as a model for pathophysiological and pharmacological research. Inflammatory stimuli have been shown to induce a rapid release of EPCs into the circulation in humans (12). In this study LPS was hypothesized to directly injure EPCs, as well as influence the number and function of EPCs, thereby influencing the endothelium repair process and disturbing the balance between the injury and repair in ALI. To evaluate this hypothesis, the number and activity of EPCs exposed to LPS *in vitro* and *in vivo* were measured.

## Materials and methods

### Animals

Male CD-1 mice (4–8 weeks old, 20–24 g) were obtained from the Laboratory Animal Center at the Nanjing Medical University (Nanjing, China). CD-1 mice were used as they are the most susceptible strains to LPS-induced injury (13). All animals in the study were inbred, thus, of the same genetic background. Animals were raised and used in accordance with the National Institutes of Health Guidelines on the Use of Laboratory Animals. All experimental procedures performed were approved by the Jinling General Hospital Committee on Animal Care (Nanjing, China).

### Isolation and culture of EPCs

EPCs were isolated from mouse bone marrow. Briefly, mononuclear cells were separated from the tibia and femur of male CD-1 mice (age, 4–8 weeks) using density gradient centrifugation (Histopaque 1083, Sigma, St. Louis, MO, USA) and cultured on human fibronectin-coated plates (ProSpec, East Brunswick, NJ, USA) in endothelial cell growth media (EGM-2) supplemented with EGM™-2 MV SingleQuots™ (Lonza, Basel, Switzerland), mouse recombinant vascular epidermal growth factor (VEGF; 20 ng/ml), insulin-like growth factor (IGF; 4 ng/ml; ProSpec), epidermal growth factor (EGF; 20 ng/ml; ProSpec) and fibroblast growth factor (FGF; 4 ng/ml; ProSpec). The EGM-2 MV SingleQuots contained: hydrocortisone, R^3^-IGF-1, human endothelial growth factor, VEGF, human FGF-B, ascorbic acid, gentamicin amphotericin-B (GA-1000) and 5% fetal bovine serum. Following four days in culture, non-adherent cells were removed by washing with phosphate-buffered saline (PBS) and adherent cells were incubated in fresh media for a further three days for the following experiments.

### Identification of EPCs

Identification of EPCs was performed using direct fluorescent staining to detect dual binding of fluorescein isothiocyanate-labeled Ulex europaeus agglutinin (FITC-UEA-1; Sigma) and 1,1-dioctadecyl-3,3,3,3-tetramethylindocarbocyanine labeled acetylated low-density-lipoprotein (Dil-ac-LDL; Molecular Probes^®^, Grand Island, NY, USA). EPCs were cultured for seven days and then incubated with Dil-ac-LDL (10 ug/ml) at 37°C for 4 h and fixed with 2% paraformaldehyde for 15 min. Cells were washed with PBS for 30 min, prior to being treated with UEA-1 (10 ug/ml) for 1 h. Cells were then washed with PBS and viewed using an inverted fluorescent microscope (IX-71, Olympus, Tokyo, Japan). Cells demonstrating double-positive fluorescence were identified as EPCs. EPCs were further identified by investigating the expression of CD34, CD133 and VEGF receptor-2 (VEGFR-2) using flow cytometry (BD Biosciences, Franklin Lakes, NJ, USA).

### EPC colony-forming unit (CFU) assay

ALI was induced in mice by administering 2.5 mg/ml LPS into the trachea (0.5 ml/kg body weight). LPS was obtained from *Escherichia coli* (O55:B5; Sigma). Following treatment with LPS, EPCs were harvested after 4, 8, 12, and 24 h, and cultured in a fibronectin-coated 24-well plate for seven days in EGM-2, as mentioned above. The control group was treated with PBS. Endothelial CFUs, characterized by a central cluster surrounded by spindle-shape cells, were counted by three independent individuals.

### Proliferation of EPCs

Monocytes from mice were seeded in a 96-well plate and cultured for seven days, the early EPCs were harvested and the EPCs were treated with various concentrations of LPS (10 pg/ml, 100 pg/ml, 1 ng/ml, 10 ng/ml and 100ng/ml) for 4, 8, 12, and 24 h. The control cells were treated with PBS. The EPCs were then analyzed using the cell counting kit-8 (CCK-8; Dojindo Laboratories, Kunamoto, Japan) in order to assess cell proliferation. CCK-8 solution was added to each well and the cells were incubated for 1.5 h. The optical density (OD) values were read using a microplate reader (Bio-Rad, Hercules, CA, USA). In the *in vivo* study, CD-1 mice were induced by the administration of 2.5 mg/ml LPS into the trachea and, after 4, 8, 12, and 24 h, the monocytes were isolated and cultured for seven days. The control group were treated with PBS. The proliferation assay was then performed in accordance with the aforementioned method.

### Senescence rate of EPCs

Cellular aging was determined using a Senescence Cell Staining kit (Sigma). In the *in vitro* study, EPCs were seeded in a 24-well plate and cultured for seven days, prior to being treated with various concentrations of LPS (control group was treated with PBS) for 4, 8, 12, and 24 h. EPCs were washed twice with PBS, prior to being fixed in fixation solution for 7 min, washed a further two times with PBS and then incubated for 12 h with fresh staining solution at 37°C without CO_2_. Following staining for 12 h, green-stained cells and total cells were counted and the percentage of β-galactosidase-positive cells was calculated. In the *in vivo* study, ALI was induced by the administration of 2.5 mg/ml LPS into the trachea. After 4, 8, 12 and 24 h EPCs were isolated and cultured for seven days. The control group was treated with PBS. The senescence assay was then conducted as mentioned above.

### Adhesion of EPCs

The adhesion assay was performed as previously described (14). Briefly, the early EPCs were cultured in three wells of a 24-well plate and were treated with various concentration of LPS (control group was PBS) for 4, 8, 12, and 24 h. Cells were then washed twice with PBS and detached with 0.5 Mm EDTA (Gibco-BRL, Grand Island, NY, USA). The EPCs were then placed in a 15-ml centrifuge tube. Cells were centrifuged at 1,000 × g (Dingguo, Beijing, China) for 5 min prior to the supernatant being removed and the EPCs being resuspended in PBS. EPCs (~2×10^4^ cells) were placed on two fibronectin-coated wells in a 24-well plate in EGM-2 and incubated for 30 min at 37°C with 5% CO_2_. Following a 30-min incubation, EPCs were washed three times with PBS, prior to being stained using Dil-ac-LDL and UEA-1 direct fluorescent staining in accordance with the aforementioned method. The adherent cells were counted by independent blinded investigators. In the *in vivo* study, ALI was induced by the intratracheal administration of LPS (2.5 mg/ml). After 4, 8, 12, and 24 h EPCs were isolated and cultured for seven days. The control group was administered PBS. The adhesion assay was performed as mentioned above.

### Statistical analysis

All experiments were performed at least six times, and the average result was calculated. Results are presented as the mean ± standard error of the mean. Student’s t-test was utilized for the comparison between two groups. Data were analyzed using Sigma Plot software (Systat Software Inc., San Jose, CA, USA). P<0.05 was considered to indicate a statistically significant difference.

## Results

### Cell morphology

**Figure 1:**
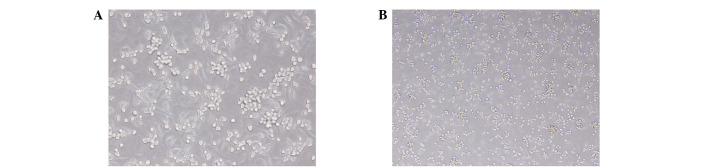
After seven days in culture, adherent cells exhibited a spindle-shaped, endothelial cell-like morphology and were identified as early endothelial progenitor cells. Colony-forming units are clearly observed. (A) Magnification, ×100; (B) magnification, ×200.

**Figure 2:**
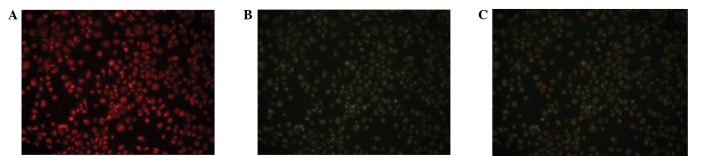
Monocytes were cultured for seven days, and adherent cells were then stained with (A) 1,1-dioctadecyl-3,3,3,3-tetramethylindocarbocyanine-labeled acetylated low-density-lipoprotein (Dil-ac-LDL; red; magnification, ×200) and (B) flourescein isothiocyanate-labeled Ulex europaeus agglutinin (FITC-UEA-1; green; magnification, ×200) and observed using inverted fluorescent microscopy. (C) Double-positive cells were identified as endothelial progenitor cells (EPCs; magnification, ×200).

Total monocytes were isolated from the bone marrow of male CD-1 mice and cultured for seven days. The resulting cells exhibited spindle-shaped, endothelium-like morphology, and were identified as early EPCs (Fig. 1). EPCs were characterized as double-positive for Dil-ac-LDL and FITC-UEA-1 staining, which was observed using inverted fluorescent microscopy (Fig. 2). EPCs also exhibited a number of other endothelial characteristics, including expression of CD34, von Willebrand factor (vWF) and VEGFR-2 (data not shown).

### CFU assay

**Figure 3:**
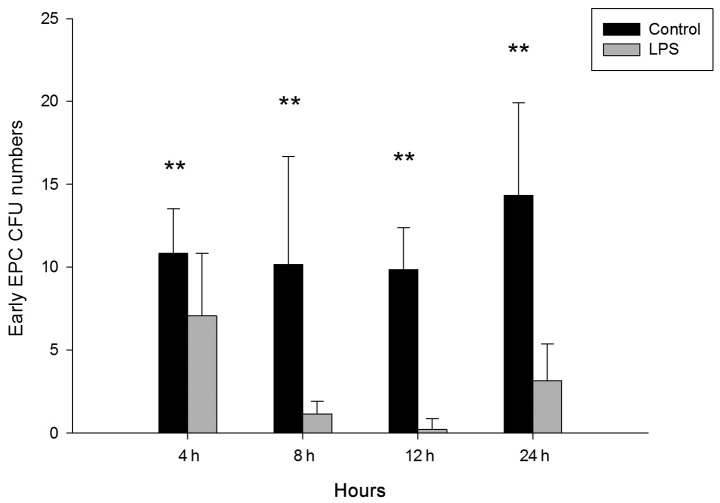
Effect of lipopolysaccharide (LPS) on endothelial progenitor cell (EPC) colony-forming units (CFUs) *in vivo*. Mice were administered LPS (2.5mg/kg) via the trachea. Control groups were treated with phosphate-buffered saline. After 4, 8, 12 and 24 h early EPCs were cultured in a fibronectin-coated 24-well plate for seven days in endothelial cell growth media; CFUs are shown. The early EPC CFUs were counted in six randomly observed visual fields (magnification, ×200). ^**^P<0.01 compared with the control group (n=6).

To investigate whether LPS influences the number of CFUs of cultured EPCs from bone marrow in mice, LPS (2.5 mg/ml) was administered via the trachea. After 4, 8, 12, and 24 h, monocytes were isolated and cultured for seven days, and early EPCs were harvested for analysis. As shown in Fig. 3, the number of CFUs was significantly reduced in the LPS-treated group compared with the control group at 4 h (control vs. LPS, 10.833±2.691 vs. 7.071±3.772; P<0.01), 8 h (control vs. LPS, 10.167±6.494 vs. 1.167±0.753; P<0.01), 12 h (control vs. LPS, 9.856±2.545 vs. 0.2222±0.667; P<0.01) and 24 h (control vs. LPS, 14.333±5.574 vs. 3.167±2.229; P<0.01).

### Proliferation of EPCs

**Figure 4:**
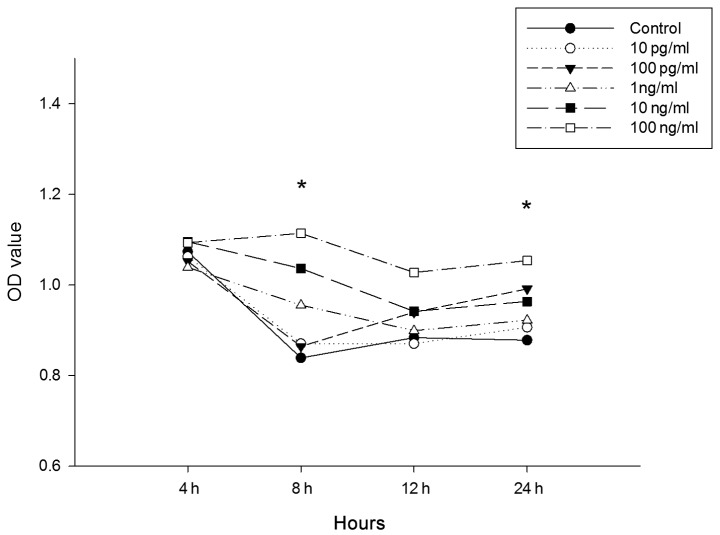
Effect of lipopolysaccharide (LPS) on endothelial progenitor cell (EPC) proliferation *in vitro*. Early EPCs were incubated with varying concentrations of LPS (10 pg/ml, 100 pg/ml, 1 ng/ml, 10 ng/ml and 100ng/ml) for 4, 8, 12, and 24 h in fibronectin-coated 96-well plates. Control cells were incubated with phosphate-buffered saline (PBS). EPC proliferation was determined using the cell counting kit-8 assay. ^**^P<0.01 compared with the control group (n=6). OD, optical density.

**Figure 5:**
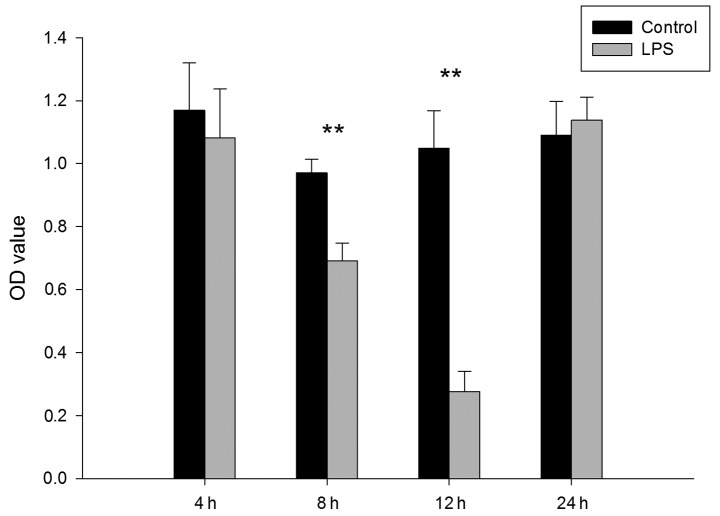
Effect of lipopolysaccharide (LPS) on endothelial progenitor cell (EPC) proliferation *in vivo*. LPS (2.5mg/kg) was administered to mice via the trachea. Control cells were treated with phosphate-buffered saline. After 4, 8, 12, and 24 h early EPCs were cultured in fibronectin-coated 96-well plates for seven days in endothelial cell growth media. EPC proliferation was determined using the cell counting kit-8 assay. ^**^P<0.01 compared with the control group (n=6). OD, optical density.

To investigate the possibility that LPS influences the proliferation of the cultured EPCs from bone marrow in mice, the CCK-8 assay was used. EPCs were cultured for seven days prior to incubation with increasing concentrations of LPS (10 pg/ml, 100 pg/ml, 1 ng/ml, 10 ng/ml and 100ng/ml) for 4, 8, 12, and 24 h. Cells treated with 10 pg/ml, 100 pg/ml and 1 ng/ml, showed no significant difference in EPC proliferation at all time-points (Fig. 4). For cells treated with 10 ng/ml for 8 h (control vs. LPS, 0.839±0.046 vs. 1.036±0.107; P<0.01; Fig. 4) and cells treated with 100 ng/m for 8 h (control vs. LPS, 0.839±0.046 vs. 1.154±0.047; P<0.01; Fig. 4) and 24 h (control vs. LPS, 0.878±0.091 vs. 1.053±0.068; P<0.01; Fig. 4), there was an increase in EPC proliferation. As shown in Fig. 5, early EPC proliferation decreased significantly at 8 h (control vs. LPS, 0.970±0.043 vs. 0.691±0.056; P<0.01) and 12 h (control vs. LPS, 1.048±0.120 vs. 0.275±0.065; P<0.01) following administration of LPS (2.5 mg/kg) via the trachea.

### Senescence of EPCs

**Figure 6:**
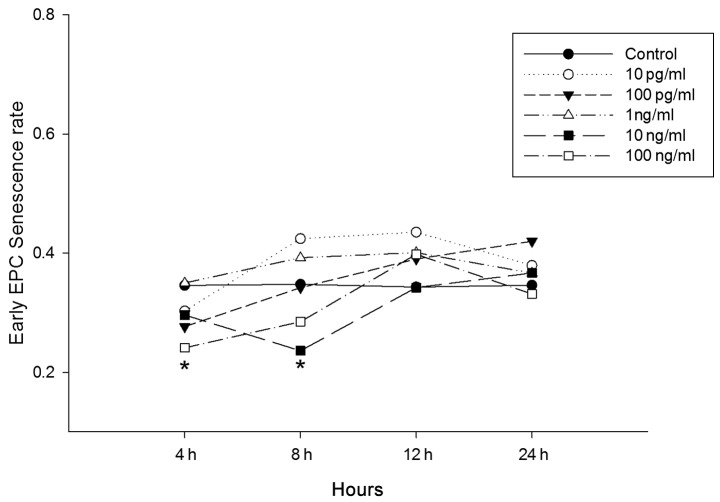
Effect of lipopolysaccharide (LPS) on endothelial progenitor cell (EPC) senescence *in vitro*. Early EPCs were incubated with varying concentrations of LPS (10pg/ml, 100pg/ml, 1ng/ml, 10ng/ml and 100ng/ml) for 4, 8, 12 and 24 h. Control cells were treated with phosphate-buffered saline, the EPC senescence cell assay was performed using a Senescence Cell Staining kit (Sigma, St. Louis, MO, USA). The early EPC senescence rates were counted in six randomly observed visual fields (magnification, ×200). ^*^P<0.05 compared with the control group (n=6).

**Figure 7:**
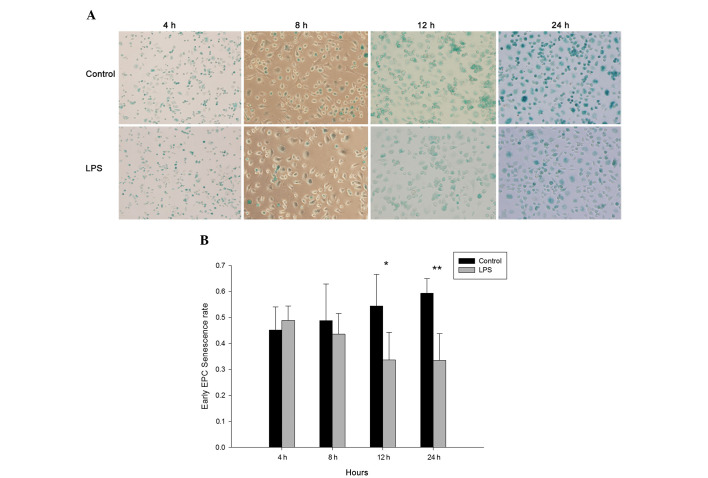
Effect of lipopolysaccharide (LPS) on endothelial progenitor cell (EPC) senescence *in vivo* (A, magnification, ×200). Mice were administered LPS (2.5mg/kg) via the trachea. Control groups were treated with phosphate-buffered saline. After 4, 8, 12 and 24 h early EPCs were cultured in a fibronectin-coated 24-well plate for seven days in endothelial cell growth media, and a senescence cell assay was performed using a Senescence Cell Staining kit (Sigma, St. Louis, MO, USA). The early EPC senescence rates were counted in six randomly observed visual fields (magnification, ×200). (B) ^*^P<0.05 and ^**^P<0.01 compared with the control group (n=6).

**Figure 8:**
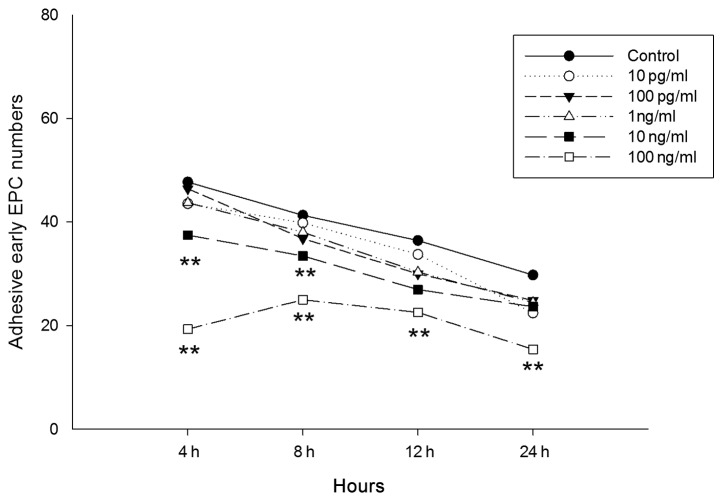
Effect of lipopolysaccharide on endothelial progenitor cell (EPC) adhesiveness *in vitro*. Early EPCs were incubated with varying concentrations of LPS (10pg/ml, 100pg/ml, 1ng/ml, 10ng/ml, 100ng/ml) for 4h, 8h, 12h, and 24h. Control cells were incubated with phosphate-buffered saline (PBS). The EPC adhesiveness assay was performed and the adhesive early EPCs were counted in six randomly observed visual fields (magnification, ×200). ^**^P<0.01 compared with the control cells (n=6).

To investigate the possibility that LPS influences the senescence of cultured EPCs from bone marrow *in vitro* in mice, EPCs were cultured for seven days prior to incubation with increasing concentrations of LPS for 4, 8, 12, and 24 h. No significant differences were observed at any time-points for cells incubated with 10 pg/ml, 100 pg/ml and 1 ng/ml LPS. Cells cultured with 10 ng/ml LPS, however, showed a significant decrease in senescence 8 h following LPS administration (control vs. LPS, 0.348±0.051 vs. 0.236±0.079; P<0.05; Fig. 6). In cells treated with 100 ng/ml LPS, the decrease was significant at 4 h (control vs. LPS, 0.346±0.083 vs. 0.241±0.045; P<0.05; Fig. 6) and 8 h (control vs. LPS, 0.348±0.061 vs. 0.285±0.057; P<0.05; Fig. 6) following LPS treatment. To investigate the influence of LPS on the senescence rate of EPCs in mice *in vivo*, LPS (2.5 mg/kg) was administered via the trachea. After 4, 8, 12, and 24 h, monocytes were isolated and cultured for seven days, and the senescence cell assay performed. As shown in Fig. 7, the rate of senescence was significantly reduced after 12 h (control vs. LPS, 0.544±0.122 vs. 0.336±0.107; P<0.05) and 24 h (control vs. LPS, 0.593±0.056 vs. 0.335±0.103; P<0.01).

### Adhesion of EPCs

**Figure 9:**
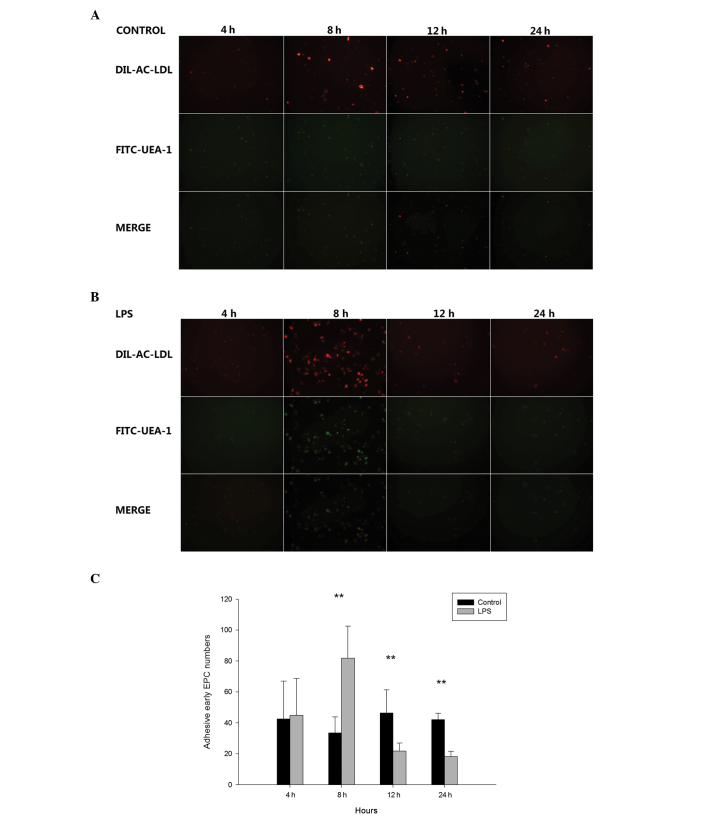
(A and B) Adhesive endothelial progenitor cells (EPCs) were identified by double staining with 1,1-dioctadecyl-3,3,3,3-tetramethylindocarbocyanine-labeled acetylated low-density-lipoprotein (Dil-ac-LDL) and fluorescein isothiocyanate-labeled Ulex europaeus agglutinin (FITC-UEA-1) in various lipopolysacchardie (LPS) concentrations (magnification, ×200). Following treatment with LPS (2.5 mg/kg), early EPCs were harvested after 4, 8, 12 and 24 h by culturing in a fibronectin-coated 24-well plate for seven days in endothelial cell growth media (EGM-2). Control cells were treated with phosphate-buffered saline (PBS). An EPC adhesion assay was performed and the adhesive early EPC numbers were counted in six randomly observed visual fields (magnification, ×200). (C) ^**^P<0.01 compared with control group (n=6).

To investigate the possibility that LPS influences the adhesion of cultured EPCs from bone marrow in mice, cultured EPCs were incubated with varying concentrations of LPS (10 pg/ml, 100 pg/ml, 1 ng/ml, 10 ng/ml and 100 ng/ml) for 4, 8, 12 and 24 h, prior to being cultured on fibronectin-coated plates for 30 min. It was observed that EPCs treated with 10 and 100 ng/ml LPS exhibited a significant decrease in adhesion, with the greatest decrease observed in cells treated with 100 ng/ml (Fig. 8). However, no significant decrease in adhesion was observed in cells treated with 10 pg/ml, 100 pg/ml and 1 ng/ml. In the 10 ng/ml group, the decrease in adhesion was significant at 4 h (control vs. LPS, 47.718±4.979 vs. 37.462±2.724; P<0.01) and 8 h (control vs. LPS, 41.308±3.549 vs. 33.461±4.181; P<0.01). In the 100 ng/ml group, the decrease in adhesion was significant at 4 h (control vs. LPS, 47.718±4.979 vs. 27.000±2.062; P<0.01), 8 h (control vs. LPS, 41.308±3.549 vs. 24.974±4.586; P<0.01), 12 h (control vs. LPS 36.436±9.475 vs. 22.538±4.757; P<0.01) and 24 h (control vs. LPS, 29.769±8.258 vs. 15.410±4.183; P<0.01). To determine the effect of LPS on EPC adhesion in mice *in vivo*, LPS (2.5 mg/kg) was administered via the trachea. After 4, 8, 12, and 24 h, EPCs were isolated and cultured for seven days prior to the adhesion assay. As shown in Fig. 9, the adhesion of the cells was greatest at 8 h (control vs. LPS, 33.500±10.349 vs. 81.833±20.677; P<0.01) and then decreased at 12 h (control vs. LPS, 46.500±14.830 vs. 21.833±5.099; P<0.01) and 24 h (control vs. LPS, 42.117±4.135 vs. 18.288±3.44; P<0.01).

## Discussion

ALI is a prevalent disease, particularly in intensive care units. Previous studies have focused on cells involved in ALI, including endothelial (15), macrophage, mononuclear and natural killer T cells (16). Numerous drugs have been found to exert a protective effect against LPS-induced ALI, including matrine, which inhibits the inflammatory response (17), heme oxygenase-1, which negatively regulates the interleukin (IL)-33 and Toll-like receptor (TLR)-4-mediated inflammatory response (18), and resolvin D1 which selectively reacts with a lipoxin A4 receptor, inhibiting mitogen-activated protein kinases and the nuclear factor-κ B pathway (19).

Previous studies investigating ALI have focused on inflammatory cells, including macrophages and leukocytes. At present, studies are targeting the endothelial repair pathway, using stem cells to attenuate the organ inflammatory response. Such stem cells have included EPCs, since their identification by Asahara *et al* (5). EPCs regenerate vascular endothelial cells and maintain the integrity of the vascular endothelium (20). The number and function of circulating EPCs has been shown to be reduced in patients afflicted with certain diseases, including cerebral aneurysm (21).

LPS is also known as endotoxin and is a key molecule involved in the initiation of sepsis syndrome (22). The LPS-induced ALI model is widely used as a disease model as it is capable of stimulating the characteristics of human ALI/ARDS (23). Local exposure to LPS may induce ALI, characterized by increased levels of neutrophils, protein content and cytokines in the bronchoalveolar lavage fluid associated with the severity of the disease (24). While it is well known that LPS induces inflammation in the lung, the present study focused specifically on the direct influence of LPS on EPCs in mice.

LPS directly influences endothelial cells through a complex signaling pathway (25). EPCs are progenitor cells that transform into endothelial cells, meaning that the effect of LPS is not the same as it is for endothelial cells. Previous data have shown that EPCs are vulnerable to LPS and have demonstrated that expression of TLR4 is the basis for this vulnerability (26). In ALI, endothelial cells and other cells are injured (1). LPS damages the constructive tissue, facilitating stem-like cells, such as EPCs, to participate in tissue repair. The tissues injured by LPS, however, are not the same as those targeted by other toxins.

The present study showed that the proliferation of EPCs induced by LPS increased in 10 and 100 ng/ml LPS *in vitro*, indicating that LPS activates EPCs rather than inhibiting them. A previous study also showed that LPS treatment failed to induce EPC apoptosis, instead promoting cell EPC proliferation (27).

The characteristics of EPCs and bone marrow-derived monocytes are associated with age-related changes (28–30). In the EPC senescence assay, it was found that the senescence rate decreased in 10 and 100 ng/ml LPS *in vitro* and also at 12 and 24 h *in vivo*. Di Stefano *et al* (31) found that LPS induces the expression of procoagulant activity in EPCs, and that the effect of LPS was dose-dependent, with statistical significance achieved at 100 ng/ml, which is consistent with the present result (31).

In the present *in vivo* study, 2.5 mg/ml (0.5 ml/kg body weight) LPS was used as previously described (32). In the course of 24 h it was found that the number of CFUs was significantly reduced at all time-points, particularly at 8 and 12 h. The rate of proliferation was also reduced significantly at 8 and 12 h; however, this was not significant at 4 and 24 h. This suggests that at 8 h ALI is most severe and gradually recovers afterwards. A previous study revealed that the number of EPCs was significantly reduced in patients treated with LPS and was lowest 6 h after LPS treatment, but after 24 h the number of cells returned to baseline levels (33). This was almost consistent with the present *in vivo* experiment. However, contradictory results exist; certain evidence has suggested that the early phase of acute low-grade inflammation is associated with a decrease in peripheral EPCs (33), whilst increased numbers of circulating EPCs have been observed in patients with bacterial pneumonia prior to treatment (12). In the present *in vitro* study the increased proliferation indicated that LPS directly influences the proliferation of EPCs. The increase in proliferation peaked 8 h after treatment with LPS.

The adhesive capacity of EPCs was impaired by LPS and showed dose- and time-dependence. Adhesion was particularly reduced at an LPS concentration of 100 ng/ml for all time-points. In the adhesion assay *in vivo*, it was found that adhesion reached a peak 8 h following LPS treatment and then gradually decreased. This indicates that, directly following treatment with LPS, the adhesion of EPCs was increased in order to repair injured endothelial cells, and then reduced in order for progenitor cells to be released from the bone marrow. The decreased adhesion at 12 and 24 h in the *in vivo* study suggests that this contributes to the release of the EPCs or the precursors of EPCs from the bone marrow and the targeting of injured tissue.

The results of the *in vitro* and *in vivo* studies were distinct, possibly due to the complex internal environment of ALI induced by LPS. The *in vitro* environment was simplified whilst the *in vivo* environment was complex, with other factors, such as cytokines, hormones and inflammatory factors, having an effect. Overall, proliferation was found to decrease *in vivo*. Decreased senescence rates and increased proliferation *in vitro* promoted the number and function of EPCs for tissue repair. The exact mechanism, however, has yet to be elucidated.

In the present study, the number of the monocytes isolated from the bone marrow was found to be decreased in the LPS-treated group compared with the control group. This suggests that the reduced numbers of EPCs and CFUs observed were partly due to the reduced number of monocytes isolated from the LPS group. LPS may directly reduce the number of monocytes, but the exact mechanism is not yet known.

The present study also demonstrated that 10 and 100 ng/ml LPS are suitable for cell culture *in vitro* due to the sensitivity of EPCs to LPS. The detailed mechanism of LPS in ALI, including the mechanism underlying its effect on EPCs, requires further investigation. The proliferation and adhesion activity of the EPCs was activated in 8 h and then gradually decreased with time. The exact mechanism has yet to elucidated, but further investigation in this area may enable more effective drugs to be developed that are capable of promoting the number and function of EPCs for participation in tissue repair.
